# Identification, characterization and functional differentiation of the *NAC* gene family and its roles in response to cold stress in ginseng, *Panax ginseng* C.A. Meyer

**DOI:** 10.1371/journal.pone.0234423

**Published:** 2020-06-11

**Authors:** Qian Liu, Chunyu Sun, Jiazhuang Han, Li Li, Kangyu Wang, Yanfang Wang, Jing Chen, Mingzhu Zhao, Yi Wang, Meiping Zhang

**Affiliations:** 1 College of Life Science, Jilin Agricultural University, Changchun, Jilin, China; 2 Research Center for Ginseng Genetic Resources Development and Utilization, Changchun, Jilin, China; Chungnam National University, REPUBLIC OF KOREA

## Abstract

The *NAC* gene family is one of the important plant-specific transcription factor families involved in variety of physiological processes. It has been found in several plant species; however, little is known about the gene family in ginseng, *Panax ginseng* C.A. Meyer. Here we report identification and systematic analysis of this gene family in ginseng. A total of 89 *NAC* genes, designated *PgNAC01* to *PgNAC89*, are identified. These genes are alternatively spliced into 251 transcripts at fruiting stage of a four-year-old ginseng plant. The genes of this gene family have five conserved motifs and are clustered into 11 subfamilies, all of which are shared with the genes of the *NAC* gene families identified in the dicot and monocot model plant species, Arabidopsis and rice. This result indicates that the *PgNAC* gene family is an ancient and evolutionarily inactive gene family. Gene ontology (GO) analysis shows that the functions of the *PgNAC* gene family have been substantially differentiated; nevertheless, over 86% the *PgNAC* transcripts remain functionally correlated. Finally, five of the *PgNAC* genes, *PgNAC05-2*, *PgNAC41-2*, *PgNAC48*, *PgNAC56-1*, and *PgNAC59*, are identified to be involved in plant response to cold stress, suggesting that this gene family plays roles in response to cold stress in ginseng. These results, therefore, provide new insights into functional differentiation and evolution of a gene family in plants and gene resources necessary to comprehensively determine the functions of the *PgNAC* gene family in response to cold and other abiotic stresses in ginseng.

## Introduction

Plants often suffer from a variety of adverse environments, such as salt, drought, cold, pathogens, and insect pests, due to their sessile lifestyle [1]. These biotic and abiotic stresses directly or indirectly affect plant growth and development, thus influencing plant production. To cope with these various environmental stresses, plants have evolved a series of defense mechanisms to resile from these stresses. As the molecular switch that can activate or inactivate gene expression, transcription factors play important roles in plant response to these stresses. The *NAC* transcription factor gene family is one of the large plant-specific gene families that plays vital roles in response to adverse environmental stresses [1,2].

The *NAC* gene family is designated, after the initials of three genes, *NAM* (NO APICAL MERISTEM, petunia), *ATAF1/2* (ARABIDOPSIS TRANSCRIPTION ACTIVATION FACTOR, Arabidopsis) and *CUC2* (CUP-SHAPE COTYLEDON, Arabidopsis). The NAC domain at the N-terminus of the NAC protein is highly conserved across all the NAC proteins identified to date in plants. This domain contains five converted motifs, designated from A to E. Motif A may be involved in the formation of dimers; Motifs C and D are highly conserved and involved in DNA binding [3]; Motifs B and E are relatively variable and may be involved in the functional diversification of the *NAC* gene family. The C-terminus of the NAC protein is a highly diverged transcriptional regulatory region with transcriptional activator or repressor activity. In addition, some *NAC* transcription factors contain an α-helical transmembrane motif at their C-termini that is responsible for anchoring to plasma membrane or endoplasmic reticulum, known as the NAC membrane-bound transcription factor [4]. The *NAC* gene family widely exists in plant species [5–7]. After the first *NAC* transcription factor-encoding gene, *NAM*, was identified from petunia, numerous genes coding NAC proteins have been identified from 57 plant species, including *Arabidopsis thaliana*, *Oryza sativa*, *Zea mays*, *Nicotiana tabacum*, and so on. Nevertheless, little is known about this gene family in ginseng, *Panax ginseng* C.A. Meyer.

The *NAC* transcription factors have been shown to participate in plant response to several abiotic stresses [8–10]. For instance, *ANAC096* is a positive response element to dehydration and osmotic stress in the ABA-dependent signal transduction pathway in Arabidopsis. The mutant of this gene, *anac096*, is less sensitive to exogenous ABA, Consequently, ABA-induced stomatal closure of the mutant was impaired and water loss was increased under drought stress conditions. On the other hand, the *ANAC096*-overexpressing (*ANACO96OX*) plants showed higher sensitive to exogenous ABA and enhanced tolerance to dehydration stress [11]. NTL4 (NAC with transmembrane motif 1-like) is another stress-related NAC transcription factor identified in Arabidopsis. Leaf senescence triggered by ROS (reactive oxygen species) can help plant survival under drought condition. By stimulating ROS production, NTL4 induced leaf senescence and sustained plant survival [12]. The rice *OsNAP* gene was significantly induced by ABA and abiotic stresses. The overexpression of *OsNAP* enhanced tolerance to drought and salinity, and increased grain yields [13]. The overexpression of tomato *SlNAM1* increased the content of osmotic adjustment substance and decreased the content of H_2_O_2_ and O_2_^•-^ under low temperature stress [14]. The overexpression of *MlNAC9* gene increased the germination rate and root growth of seedlings in *Miscanthus*, and enhanced tolerance to drought and cold stresses in Arabidopsis transgenic plants [15]. Therefore, as one of the gene families that are responsible for plant response to different abiotic stresses, identification of new members of the *PgNAC* gene family that are involved in abiotic stress responses will facilitate research in the functions of the gene family in response to abiotic stresses in ginseng.

Ginseng is a perennial overwintering herb in the *Araliaceae* family. It has been historically widely used for human health and medicine for thousands of years, and has been a model species for medicinal plant research in medicinal chemistry, genetics and genomics [16]. Ginseng is vulnerable to environmental stresses, and its production and quality are significantly affected by adverse environments. Therefore, it has become the urgent issue for ginseng production and industry to improve its tolerance to these adverse environmental stresses. It has been shown that ginseng responded to environmental stresses by enhancing the expressions of defense-related genes [17]. The present study took advantages of several transcriptomes recently developed in ginseng, identified the gene members of the *NAC* gene family in ginseng, designated hereafter *PgNAC* gene family, and characterized them in several aspects. Then, we identified five genes of the family that are responsive to cold stress. The findings of this study, therefore, provide new insights into functional differentiation and evolution of the *NAC* gene family in plants and *NAC* gene resources necessary to comprehensively elucidate the functions of the *NAC* gene family in ginseng, especially in plant growth, development, and response to abiotic stresses.

## Materials and methods

### Database resources

A transcriptome database consisting of 248,993 transcripts [17], herein designated Database A, was used for this study. This database was previously generated from 14 tissues of a four-year-old plant of Jilin ginseng cv. Damaya sampled at the fruiting stage, including fiber root, leg root, main root epiderm, main root cortex, rhizome, arm root, stem, leaf peduncle, leaflet pedicel, leaf blade, fruit peduncle, fruit pedicel, fruit flesh, and seed. Moreover, two other transcriptome databases, Database B and Database C, were also used for this study. Database B was developed from the roots of 5-, 12-, 18- and 25-year-old Jilin ginseng plants and Database C developed from the four-year-old plant roots of 42 genotypes (coded from S1 to S42) of Jilin ginseng [18].

### Identification of *PgNAC* genes

Two methods were used to identify the *PgNAC* genes in ginseng from Jilin ginseng Database A [17]. The first one used the NAC domain of the NAC proteins (PF02365) (http://pfam.xfam.org) as queries to tblastn search Database A for *PgNAC* genes at E-value ≤ le-05. The second method used the NAC domain of these NAC proteins (PF02365) to establish the HMM (Hidden Markov Model) profile using the Hummer software. The protein sequences obtained by HMM were used as queries to also search Database A for *PgNAC* genes. Then, the NAC protein sequences obtained from the above two searches were combined and verified by existence of the NAC domain using NCBI CDD database (https://www.ncbi.nlm.nih.gov/Structure/cdd/wrpsb.cgi). The genes confirmed to contain the NAC domain were designated *PgNAC* genes.

### Protein conserved motifs and phylogeny of the *PgNAC* genes

Open-reading frame (ORF) search of the *PgNAC* genes was performed using the ORF Finder (http://www.ncbi.nlm.nih.gov/gorf/gorf.html). The conserved domains of the *PgNAC* genes that had complete ORFs were predicted using the NCBI CDD database (https://www.ncbi.nlm.nih.gov/Structure/cdd/wrpsb.cgi). Then, these *PgNAC* genes were subjected to motif prediction using the MEME software [19]. A neighbor joining (NJ) phylogenetic tree was constructed using MEGA 5.0 based on multiple alignment [20]. The phylogenetic tree was constructed with 10,000 bootstrap replications and visualized using the iTOL software (https://itol.embl.de/itol.cgi).

### Functional categorization and enrichment analysis of the *PgNAC* genes

The *PgNAC* genes were functionally categorized with gene ontology (GO) terms using the Blast2GO version 4.1.9 software [21]. The GO functional categorization of the transcripts of the entire Database A [17] was used as the background control or theoretical number of transcripts categorized into each subcategory (Level 2) for enrichment analysis of the *PgNAC* transcripts. Enrichment of the number of *PgNAC* transcripts categorized into each subcategory was determined by Chi-square test.

### Construction of co-expression network of the *PgNAC* genes

All 251 *PgNAC* transcripts were subjected to co-expression network analysis. The co-expression network was constructed by BioLayout Express^3D^ Version 3.2 software [22].

### Roles of the *PgNAC* gene family in ginseng response to cold stress

Ginseng is a perennial winter herb to which cold tolerance is crucial. Therefore, we further examined the roles of the *PgNAC* gene family in response to cold stress. The hairy roots of ginseng were cultured on 1/2 MS medium at 22°C in dark for 28 days and then subjected to experimental treatment. For cold stress treatment, the hairy roots were stressed at 4°C with three biological replicates. For non-cold stress control, the hairy roots were continuously cultured at 22°C, also with three biological replicates. The hairy root samples cultured at 4°C (cold treated) and 22°C (non-cold treated) were collected at 6 h, 12 h, 24 h, 48 h, and 72 h, respectively, and stored at -80°C for further analysis.

#### Malondialdehyde assay

Malondialdehyde (MDA), a product of peroxidation of polyunsaturated fatty acids in phospholipids, has been widely used as an indicator for cell membrane damage resulting from abiotic stresses, including cold stress [23–25]. Therefore, we employed the content variations of MDA in cold-treated ginseng hairy roots relative to that of non-cold-treated ginseng hairy roots to confirm efficiency of the cold stress treatment. The content of MDA was measured according to Zhu et al. [26].

#### Expressions of PgNAC genes

The collected samples of hairy roots were subjected to RT-qPCR, with three biological and three technical replicates, to estimate the expressions of the *PgNAC* genes that were likely involved in ginseng response to abiotic stresses, including cold stress (for the *PgNAC* genes, see Results), in the cold-treated hairy roots relative to in the non-cold-treated hairy roots, Total RNA was extracted using the TRIzol reagent (Invitrogen) according to manufacturer's instructions (BioTeke Corporation). Reverse transcription was performed using a commercial kit (Beijing ComWin Biotech). *GADPH* was used as a reference gene. The RT-qPCR reactions were carried out with a pre-denaturation step at 95°C for 10 min, followed by 40 cycles of denaturation (95°C for 15 s), annealing (60°C for 1 min), and extension (68°C for 10 s), and a final stage of 60°C - 95°C to determine the melting curves of the amplified products. The relative expression of a target gene was determined by the 2^-ΔΔCt^ method [27].

## Results

### Identification and number variation of *PgNAC* genes

A total of 251 transcripts containing partial or complete NAC domains were obtained. These transcripts were alternatively spliced from 89 *PgNAC* genes. These 89 *PgNAC* genes were designated *PgNAC01* to *PgNAC89*, with different transcripts derived from a *PgNAC* gene suffixed with “-01, -02…” (S1 Table). The nucleotide sequences of these 251 transcripts had a length ranging from 202 bp to 7,965 bp, with an average length of 1,627 bp.

When the 251 transcripts were aligned to three ginseng databases, with criteria of alignment length ≥ 200 nucleotides or amino acids, identity ≥ 90%, and e-value ≤ 1.0E-06, 105 (42%) of them were aligned to the ginseng cv. ChP genome [28], 116 (46%) aligned to the ginseng cv. ChP *NAC* gene database [29], and 111 (44%) aligned to the Strain IR826 protein database [30] (S2 Table). One hundred seventeen (47%) of them that were spliced from 55 (61.8%) of the 89 *PgNAC* genes identified in this study were aligned to none of the three ginseng databases, suggesting that they were highly likely specific for the genotype, Jilin ginseng cv. Damaya, analyzed in this study. This result also suggested that the number of genes in the *PgNAC* gene family varied among genotypes within *P*. *ginseng*. This discrepancy could be attributed to the variation in number of the *PgNAC* genes among genotypes, because it has been documented that the number of genes in a gene family or a genome varied dramatically among genotypes within a species [26, 31, 32].

### Sequence analysis and motif identification

**Fig 1:**
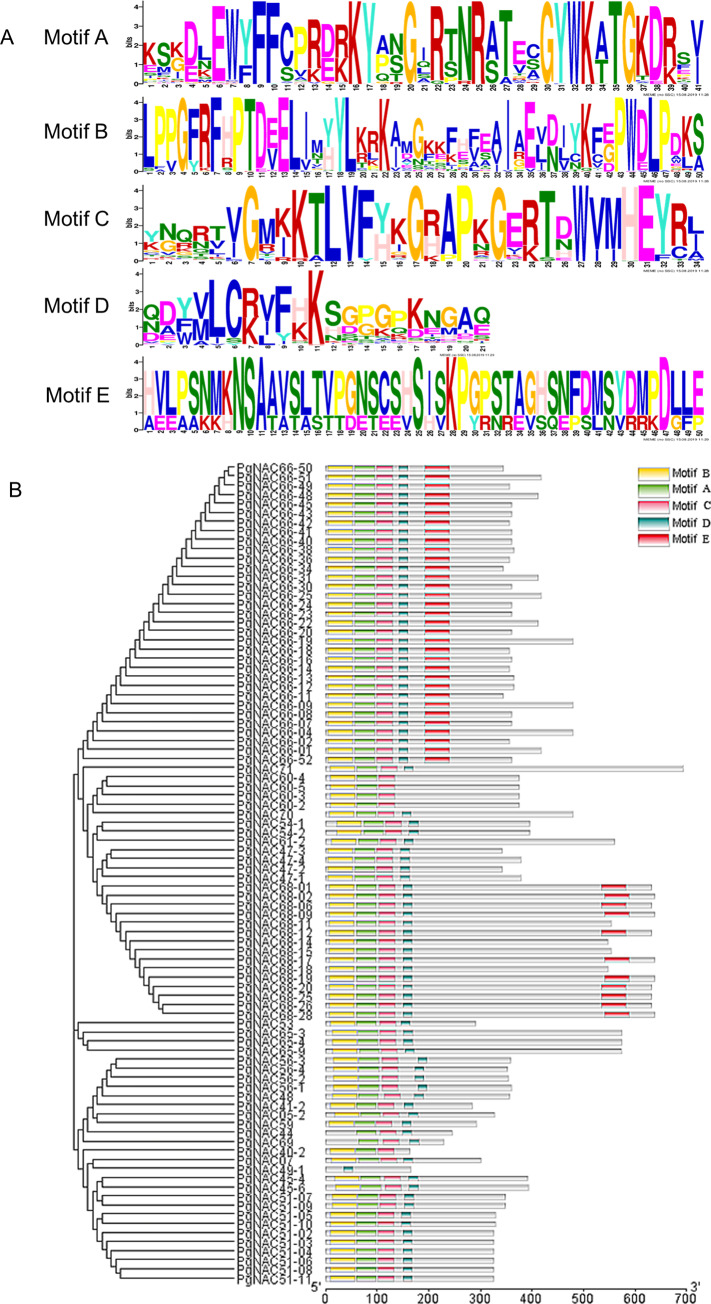
The conserved motifs (A) and their distributions (B) in *PgNAC* putative proteins.

ORF analysis showed that 90 (35.9%) of the 251 transcripts that derived from 22 of the 89 *PgNAC* genes had complete NAC domains within ORFs. Therefore, these 90 *PgNAC* transcripts were used for motif identification. Five conserved motifs, A, B, C, D and E, were found (Fig 1A). The N-termini of the *PgNAC* putative proteins were highly conserved, suggesting that the conservation may be essential for the function of the NAC proteins. Of the 90 *PgNAC* putative proteins, 44 (48.9%) contained all five motifs; 33 (36.7%) contained four of the five motifs, A, B, C, and D; 6 (6.7%) contained three motifs, A, B, and C; 3 (3.3%) contained three motifs, B, C, and D; 3 (3.3%) contained two motifs, B and C; and 1 (1.1%) contained one motif, D (Fig 1B). Except for *PgNAC49-1* whose putative protein only contained Motif D, the putative proteins of all remaining 89 *PgNAC* transcripts contained both Motifs B and C. Compared with Motifs B and C, Motif E was more variable. The results showed that Motifs B and C of the *PgNAC* putative proteins were highly conserved and shared with the NAC proteins of other species [33–35].

### Phylogenetic analysis of the *PgNAC* gene family

**Fig 2:**
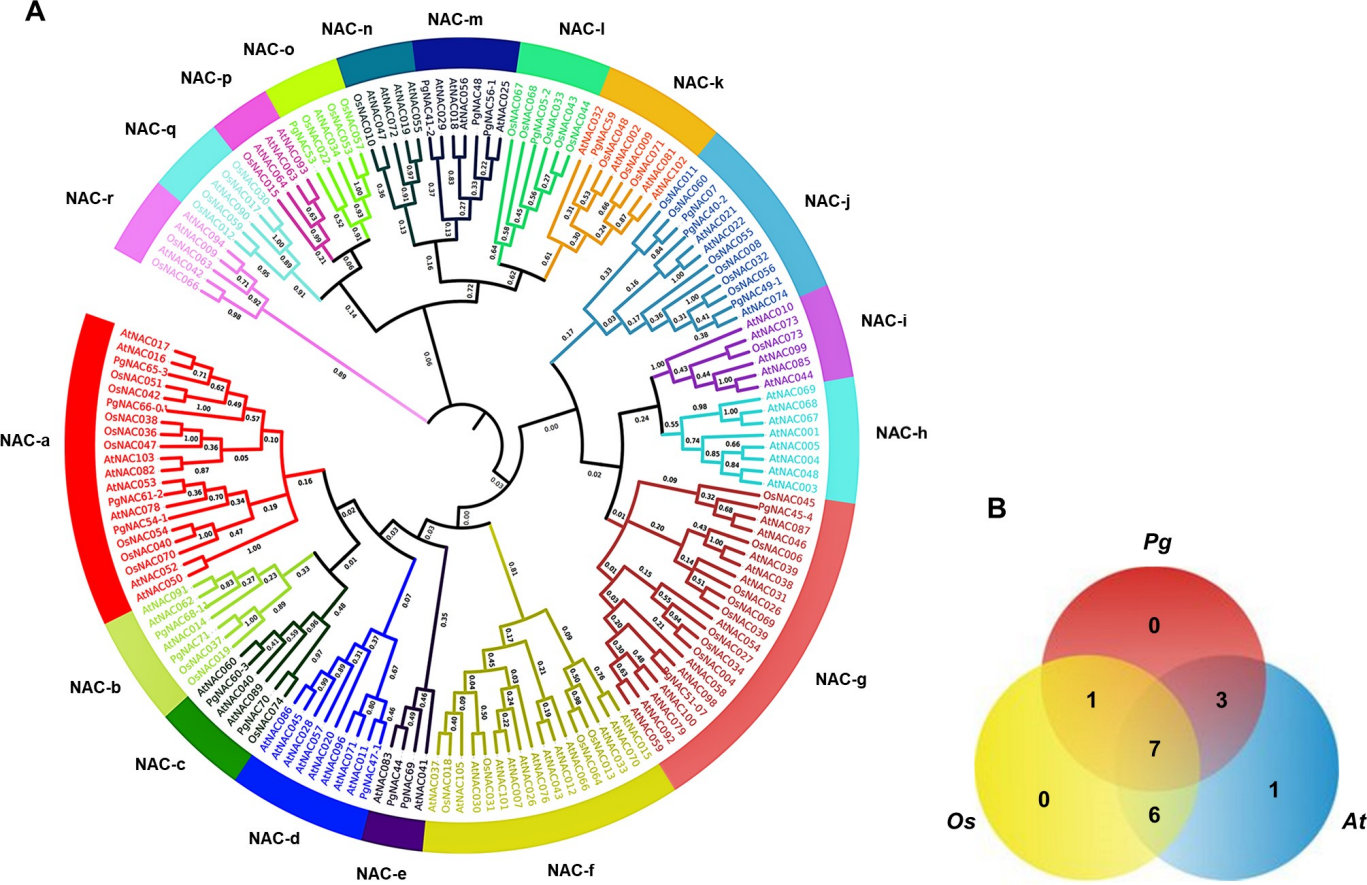
Phylogenetic tree of the *PgNAC* gene family. (A) The neighbor-joining (NJ) phylogenetic tree of the *PgNAC* genes with the *AtNAC* genes in *Arabidopsis thaliana* (*At*) and *OsNAC* genes in rice (*Oryza sativa*, *Os*). The subfamilies of the *NAC* gene families are defined from NAC-a through NAC-r. The tree was constructed with 10,000 bootstrap replications. (B) Comparison of the subfamilies among the *PgNAC*, *AtNAC* and *OsNAC* gene families. The numbers in the Venn diagram indicate the numbers of subfamilies shared among the three *NAC* gene families and the number of subfamilies specific for each species.

To determine the phylogeny of the *PgNAC* gene family, one *PgNAC* transcript that had the longest sequence and/or the largest number of conserved motifs were selected for each *PgNAC* gene. As a result, 22 *PgNAC* transcripts that were representative for 22 *PgNAC* genes were selected and used to construct the phylogenetic tree of the *PgNAC* gene family. Eighty-six *AtNAC* genes from *Arabidopsis thaliana* (*At*) and 50 *OsNAC* genes from *Oryza sativa* (*Os*) were used as evolutionary controls (S3 Table). The selected transcripts of the 22 *PgNAC* genes were translated into putative proteins, aligned and constructed into the phylogenetic tree of the *PgNAC* gene family (Fig 2A). The *PgNAC* genes were classified into 11 subfamilies, while the *AtNAC* genes and *OsNAC* genes were classified into 17 and 14 subfamilies, respectively. Of the 11 *PgNAC* gene subfamilies, 7 (63.6%) were classified with both the *AtNAC* genes and the *OsNAC* genes, 10 (90.9%) with the *AtNAC* genes, and 8 (72.7%) with the *OsNAC* genes (Fig 2B). No *PgNAC* gene was classified into a new subfamily. This result indicated that the *PgNAC* gene family is an ancient gene family originated before the divergence between the monocotyledon (rice) and dicotyledon (Arabidopsis and ginseng) plants. Nevertheless, the evolution of the *PgNAC* gene family had actively occurred after the separation of the dicotyledon (Arabidopsis and ginseng) plants from the monocotyledon (rice) plants because the *PgNAC* genes shared more subfamilies with the *AtNAC* genes than with the *OsNAC* genes. No significant evolution of the *PgNAC* gene family occurred afterward.

The phylogenetic analysis also showed that some of *PgNAC* gene putative proteins were highly homologous with those of *AtNAC* and *OsNAC* genes, such as *PgNAC61-2* with *AtNAC053*, *PgNAC47-1* with *AtNAC011*, *PgNAC44* with *AtNAC083*, *PgNAC51-07* with *AtNAC098*, *PgNAC07* with *OsNAC060*, *PgNAC49-1* with *AtNAC074*, *PgNAC41-2* with *AtNAC029*, *PgNAC56-1* with *AtNAC025*, *PgNAC53* with *AtNAC036*, and *PgNAC60-3* with *AtNAC060* (Fig 2A). These results indicated that these *PgNAC* genes may have the same or similar functions in ginseng to those in Arabidopsis and/or rice.

### Functional differentiation of the *PgNAC* gene family

**Fig 3:**
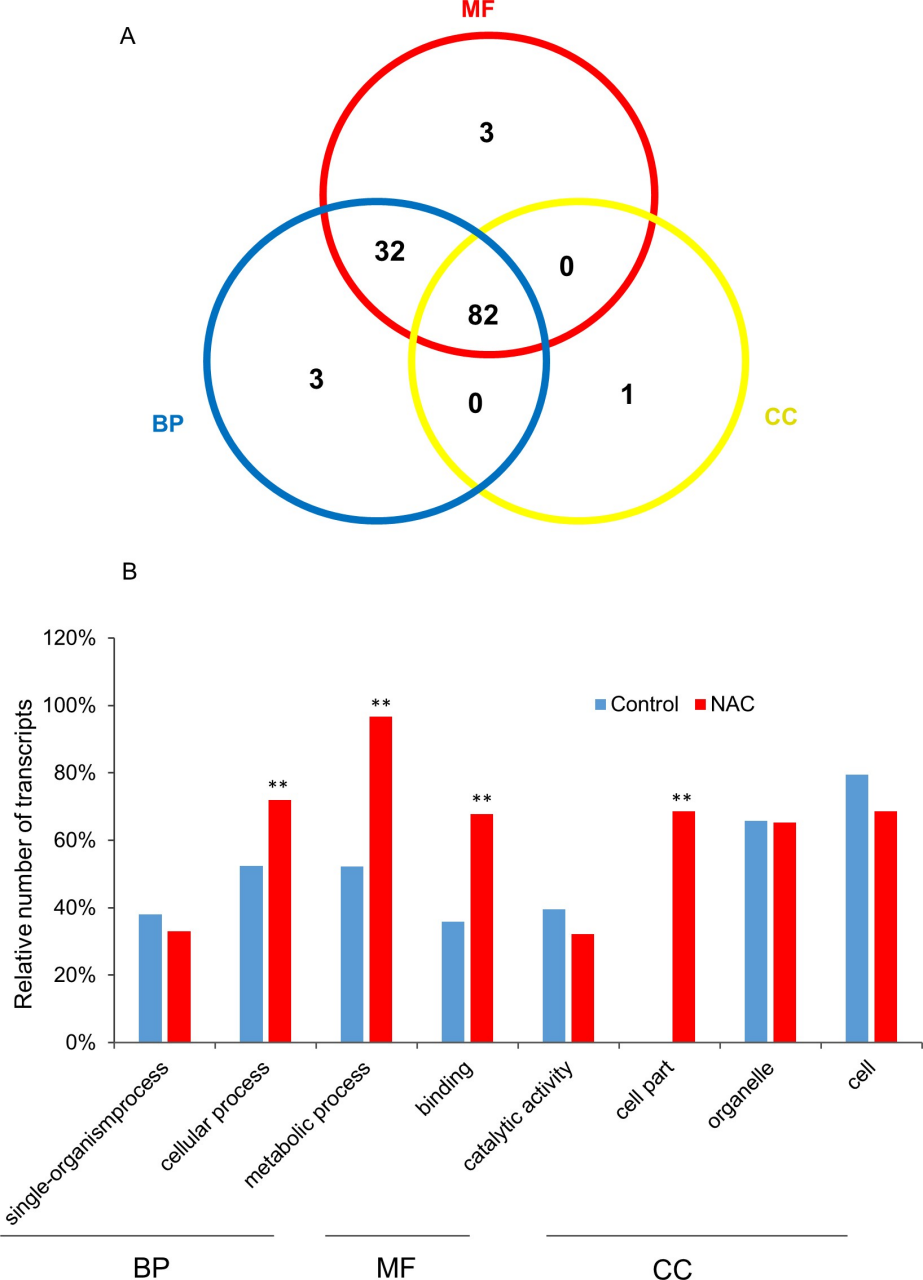
Functional categorization of the *PgNAC* transcripts. (A) Venn diagram of the primary functional categorization of the *PgNAC* transcripts. BP, biological process; MF, molecular function; and CC, cellular component. (B) functional categorization of the *PgNAC* transcripts at Level 2 and their enrichments. The GO terms of the transcripts expressed in the 14 tissues of a four-year-old plant were used as the background control for the enrichment analysis. **, significant at *P* ≤ 0.01.

The gene members in a gene family, because they share high sequence identity and/or the same conserved domain(s), have been often considered to have the same or similar functions. Nevertheless, recent studies showed that the functions of the genes in a gene family have been dramatically differentiated, indicating that they may not have same or similar functions [26, 36, 37]. To understand the functional differentiation of the *PgNAC* gene family, the 251 *PgNAC* transcripts were annotated and functionally categorized. Only 121 (48%) of the 251 *PgNAC* transcripts were annotated, while the remaining 130 could not be annotated, suggesting the uniqueness of the *PgNAC* genes in ginseng. The annotated *PgNAC* transcripts were categorized into all three primary functional categories: biological process (BP), molecular function (MF) and cellular component (CC). Of the 121 transcripts annotated, 82 were categorized into all three primary categories (67.8%), MF, BP and CC; 32 (26.5%) into the BP and CC categories; and 7 (5.8%) into one of the three primary categories (Fig 3A). Of these seven transcripts, *PgNAC67-2*, *PgNAC67-3*, and *PgNAC81* were categorized into BP; *PgNAC63-78*, *PgNAC63-16*, and *PgNAC63-6* categorized into MF; and *PgNAC10* categorized into CC (Fig 3A; S4 Table).

At Level 2, the 121 *PgNAC* transcripts were categorized into eight subcategories, including single-organism process (33.1%), cellular process (71.9%), metabolic process (96.7%), binding (67.8%), catalytic activity (32.2%), cell part (68.6%), organelle (65.3%), and cell (68.6%) (Fig 3B; S4 Table). Of the eight subcategories, the number of the *PgNAC* transcripts were significantly up-enriched, relative to the background control, in the cell process, metabolic process, binding, and cell part subcategories. These results indicated that the functions of *PgNAC* genes have substantially differentiated, ever though they have high sequence identity and all contain the NAC domain, further supporting the findings that the different genes in a gene family may not have same or similar functions [26, 36, 37].

**Fig 4:**
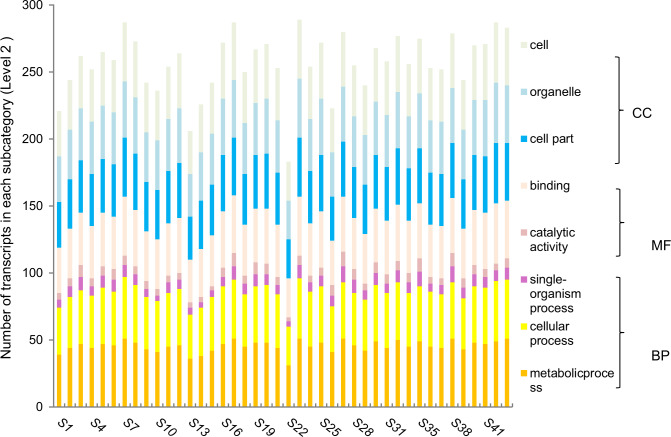
Variation of the functional categorization of the *PgNAC* transcripts among the four-year-old plant roots of 42 genotypes sampled from Jilin, China.

**Fig 5:**
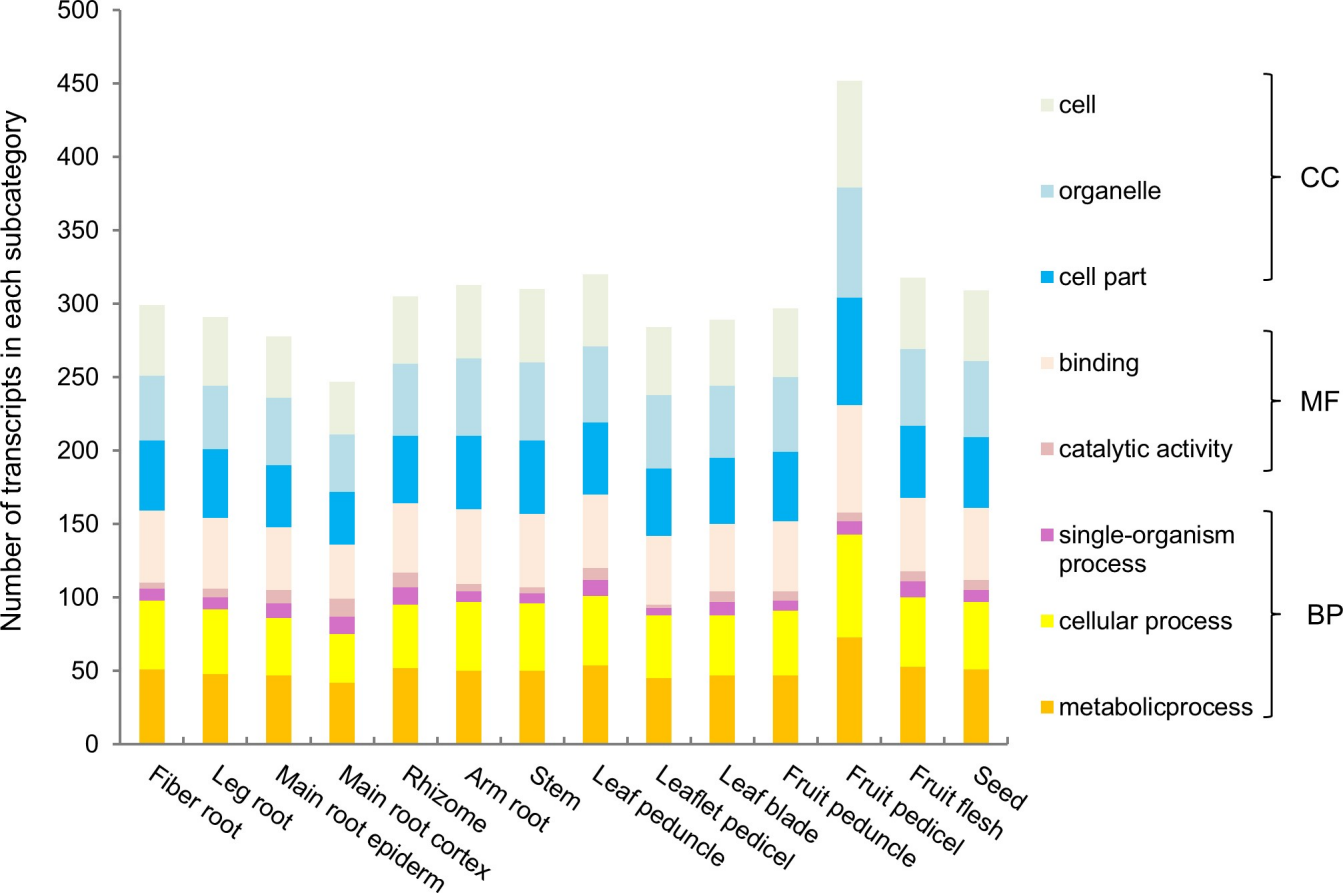
Variation of the functional categorization of the *PgNAC* transcripts among 14 tissues of a four-year-old plant.

**Fig 6:**
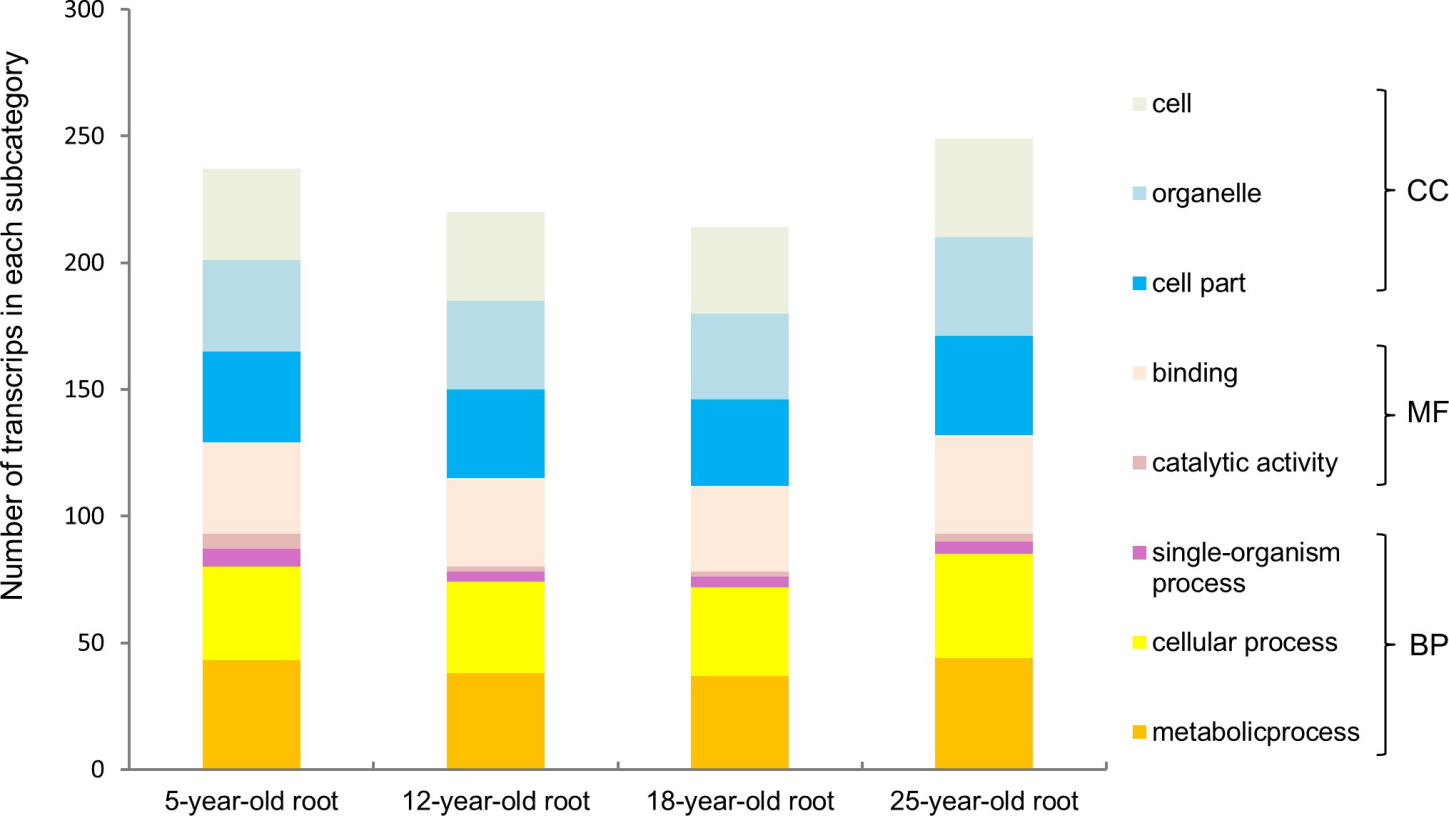
Variation of the functional categorization of the *PgNAC* transcripts among the roots of 5-, 12-, 18- and 25-year-old plants.

Furthermore, we functionally categorized the 121 annotated *PgNAC* transcripts in the four-year-old plant roots of 42 genotypes (Fig 4), in the 14 tissues of a four-year-old plant (Fig 5), and the roots of 5-, 12-, 18- and 25-year-old plants (Fig 6). Although the number of the *PgNAC* transcripts categorized into each subcategory (Level 2) substantially varied among tissues, across genotypes, and at different developmental stages, the *PgNAC* transcripts expressed in the four-year-old plant roots of 42 genotypes, the 14 tissues of a four-year-old plant, and the roots of 5-, 12-, 18- and 25-year-old plants were all categorized into the eight subcategories. These results further confirmed the functional differentiation of the *PgNAC* gene family and also demonstrated the functional consistency of the *PgNAC* gene family at different developmental stages, in different tissues and among genotypes.

### Temporal-spatial expressions of the *PgNAC* transcripts and their variation across genotypes

To characterize the expression patterns of the *PgNAC* transcripts, the expression profiles of all 251 *PgNAC* transcripts in the roots of 5-, 12-, 18- and 25-year-old plants, the 14 tissues of a four-year-old plant, and the four-year-old plant roots of 42 genotypes were quantified. The result showed that the expressions of the *PgNAC* transcripts varied from 0.00 TPM to 400 TPM temporally, spatially and across genotypes. Of the 251 *PgNAC* transcripts, 160 (63.7%) expressed in all four different year-old plant roots, 226 (90%) expressed in all 14 tissues of a four-year-old plant, and 218 (86.9%) expressed in the four-year-old roots of all 42 genotypes (S5 Table). Eight of the 251 *PgNAC* transcripts, *PgNAC63-33*, *PgNAC63-51*, *PgNAC63-56*, *PgNAC63-80*, *PgNAC66-13*, *PgNAC66-18*, *PgNAC66-36*, and *PgNAC79*, were silent at all of the developmental stages, tissues and genotypes studied (S5 Table).

### Functional relationship among the gene members of the *PgNAC* gene family

**Fig 7:**
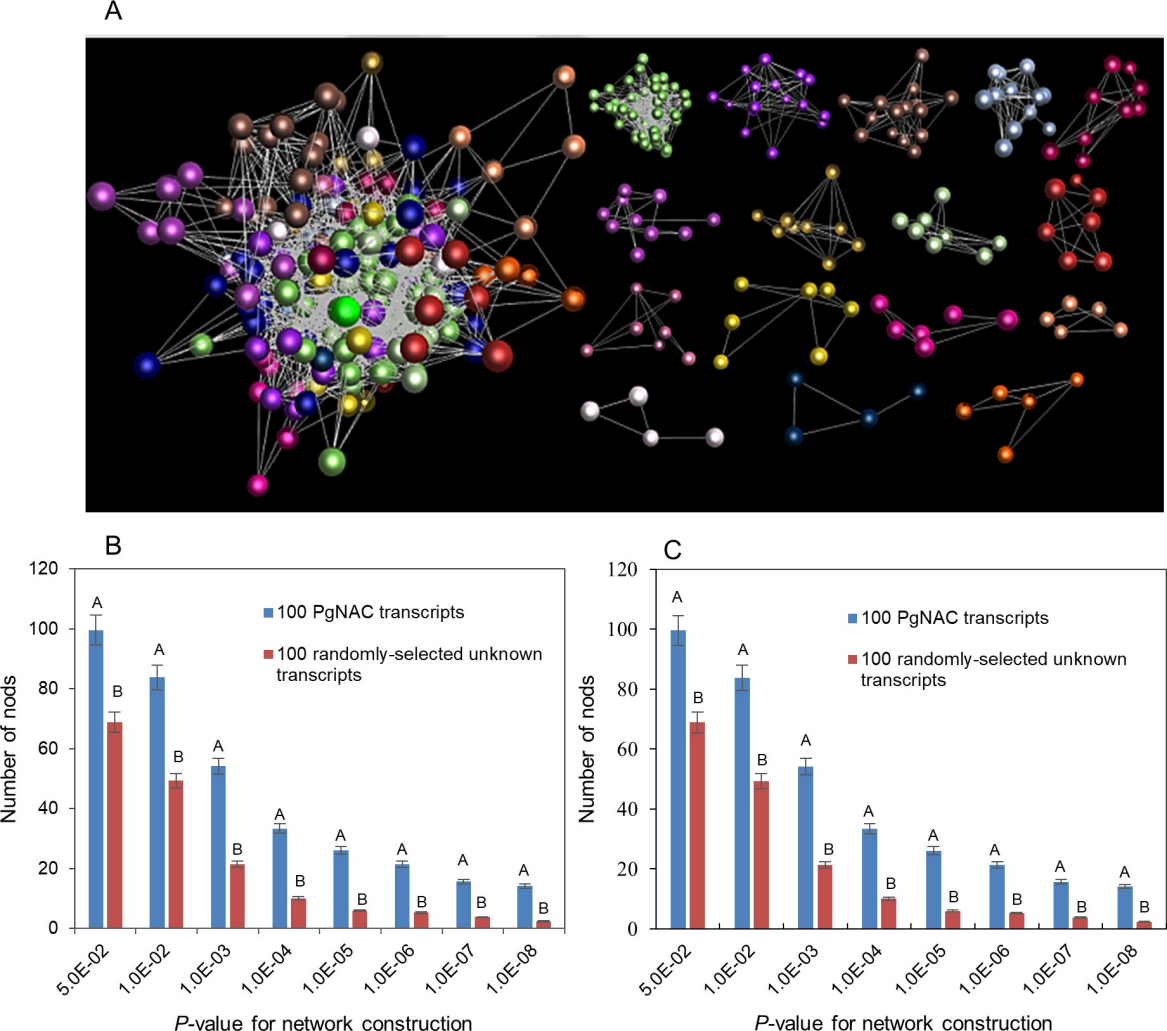
Co-expression network of the *PgNAC* transcripts in the four-year-old plant roots of 42 genotypes. These 42 ginseng genotypes were collected from the origin and diversity center of ginseng, Jilin, China. (A) The co-expression network and its clusters constructed from 218 of the 251 *PgNAC* transcripts at *P* ≤ 5.0E-02. The network consisted of 218 *PgNAC* transcript nodes and 2,245 co-expression edges. (B) Statistics of the tendency of the *PgNAC* transcripts forming a co-expression network: number of nodes. (C) Statistics of the tendency of the *PgNAC* transcripts forming a co-expression network: number of edges. The 100 *PgNAC* transcripts were randomly selected from the 251 *PgNAC* transcripts identified in this study by bootstrap sampling, with 10 replications, while the 100 randomly-selected unknown transcripts were randomly selected from Database A [17]. Different capital letters indicate that the difference is significant at a two-tailed significance of *P* ≤ 0.01.

To find out whether the gene members of the *PgNAC* gene family are functionally related, we further analyzed their co-expression networks in the four-year-old plant roots of 42 ginseng genotypes and in the 14 tissues of a four-year-old plant, respectively. As a result, 218 of these 251 *PgNAC* transcripts formed a co-expression network in the four-year-old plant roots of 42 genotypes (Fig 7A). The network consisted of 16 clusters, 218 transcript nodes and 2,245 transcript-transcript co-expression edges. In comparison, the *PgNAC* transcripts were much more likely to form a co-expression network than the transcripts randomly selected from Database A in both number of nodes (Fig 7B) and number of edges (Fig 7C).

**Fig 8:**
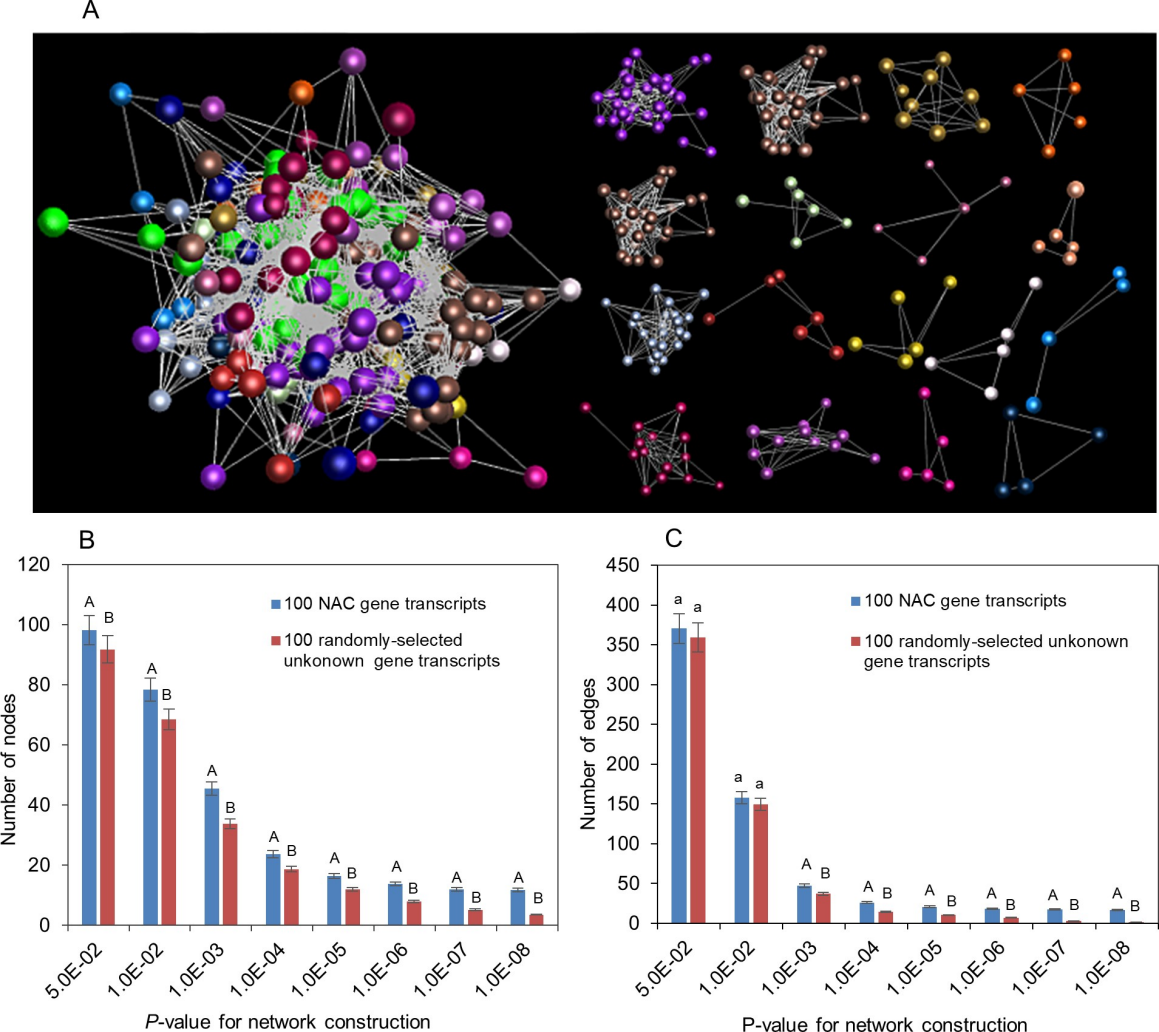
Co-expression network of the *PgNAC* transcripts in 14 tissues of a four-year-old plant. These 14 tissues were collected at the fruiting stage of the plant. (A) The co-expression network and its clusters constructed from 226 of the 251 *PgNAC* transcripts at *P* ≤ 5.0E-02. The network consisted of 226 *PgNAC* transcript nodes and 1,935 transcript co-expression edges. (B) Statistics of the tendency of the *PgNAC* transcripts forming a co-expression network: number of nodes. (C) Statistics of the tendency of the *PgNAC* transcripts forming a co-expression network: number of edges. The 100 *PgNAC* transcripts were randomly selected from the 251 *PgNAC* transcripts identified in this study by bootstrap sampling, with 10 replications, while the 100 randomly-selected unknown transcripts were randomly selected from Database A [17]. Different capital letters indicate that the difference is significant at a two-tailed significance of *P* ≤ 0.01. The same small letters indicate that the difference is not significant at a two-tailed significance of *P* ≤ 0.05.

Similarly, 226 of the 251 *PgNAC* transcripts formed a co-expression network in the 14 tissues of a four-year-old plant. The network consisted of 17 clusters, 226 transcript nodes and 1,935 transcript-transcript co-expression edges (Fig 8A). These *PgNAC* transcripts were also much more likely to form a co-expression network than the transcripts randomly selected from Database A in both number of nodes (Fig 8B) and number of edges (Fig 8C). These results indicated that the gene members of the *PgNAC* gene family functionally differentiated, but they were still functionally correlated in ginseng.

### Roles of the *PgNAC* gene family in plant response to cold stress

Studies showed that the *NAC* gene family responded to a variety of abiotic and biotic stresses in plants, including cold stress [38, 39]. Nakashima et al. [39] grouped the *NAC* genes of Arabidopsis (*A*. *thaliana*), rice (*O*. *sativa*), lycophyte (*Selaginella moellendorffii*), and moss (*Physcomitrella patens*) into six ancient groups: AM/CUC3, SND, TIP, SNAC, ANAC034, and ONAC4. Zhu et al. [4] and Nakashima et al. [39] showed that many of the *NAC* genes grouped into the SNAC group played roles in response to abiotic stresses. Of the 22 *PgNAC* genes identified in this study, five were grouped into the SNAC group (S1 Fig). Nuruzzaman et al. [9] found that the SNAC group had a highly conserved motif (WVLCR) in the region outside the NAC domain (S2 Fig). Therefore, we further investigated in this study these five *PgNAC* genes, *PgNAC05-2*, *PgNAC41-2*, *PgNAC48*, *PgNAC56-1*, and *PgNAC59*, to explore whether the *PgNAC* gene family has roles in plant response to abiotic stresses.

**Fig 9:**
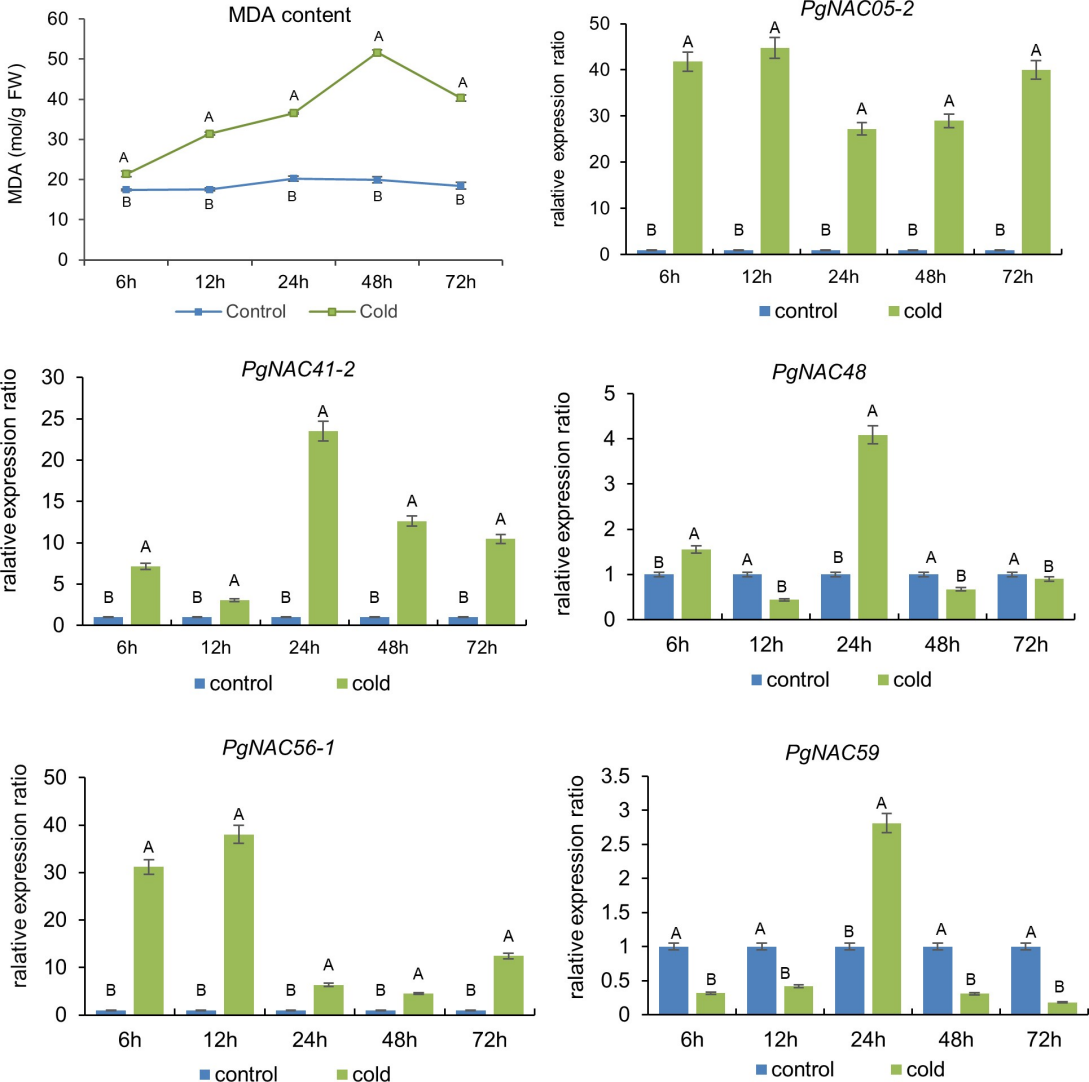
Increase of the MDA content and expression regulation of *PgNAC* transcripts induced by cold treatment in ginseng hairy roots. The experiments for both cold treatment and non-cold-treated control were carried out with three biological replicates. MDA, malondialdehyde, has been widely used as an indicator of plant response to cold stress [23–25].

Cold is one of the abiotic stresses crucial to plants, especially to winter plants, such as ginseng and winter wheat. Therefore, we investigated in this study the roles of the five *PgNAC* genes, *PgNAC05-2*, *PgNAC41-2*, *PgNAC48*, *PgNAC56-1*, and *PgNAC59*, in plant response to cold stress. Because previous studies showed that cold stress induced oxidative damage on cell membrane and MDA is an indicator of cell membrane damage, such as membrane lipid peroxidation and cell membrane permeability, due to cold stress [40], we measured the MDA contents of ginseng hairy roots, after exposed to 4°C for 6 h, 12 h, 24 h, 48 h, and 72 h, with three biological replicates. Two-tailed *t*-test showed that the contents of MDA in the cold-treated hairy roots were significantly higher than those in the non-cold treated controls (Fig 9) for each of the five time points, suggesting that the cold stress significantly increased the contents of MDA in the cold-treated ginseng hairy roots. The contents of MDA in the cold-treated roots increased along with the treatment time increase (Fig 9). The MDA content of the cold-treated hairy roots reached its peak at 48 hours after cold stress, which was nearly 3 times higher than that of the non-cold treated roots, and then decreased. These results indicated that the cold treatments were proper for assaying expression response of the *PgNAC* genes to cold stress.

Accordingly, the expression levels of all five *PgNAC* transcripts (*PgNAC05-2*, *PgNAC41-2*, *PgNAC48*, *PgNAC56-1*, and *PgNAC59*) analyzed were significantly up- or down-regulated in the cold-treated roots, relative to the non-cold-treated roots. The patterns of root response to the cold treatment varied dramatically among the five *PgNAC* transcripts studied. For instance, *PgNAC05-2* and *PgNAC56-1* reached their highest expression levels at 12 h, while *PgNAC41-2*, *PgNAC48*, and *PgNAC59* reached their expression peaks at 48 h (Fig 9). These results demonstrated that the *PgNAC* gene family was involved in plant response to cold stress, even though the expression patterns of the *PgNAC* genes varied, indicating that the *PgNAC* gene family plays roles in plant response to cold stress.

## Discussion

The *NAC* gene family has been shown to play important roles in plant response to varying environments. This study has systematically analyzed the *NAC* gene family in ginseng, designated the *PgNAC* gene family. The results reveal that the *PgNAC* gene family consists of at least 89 gene members, because they are identified from the transcriptome of 14 tissues of a four-year-old ginseng plant, and the number of the genes in the family varies dramatically among genotypes within the ginseng species. This number of the genes in the *PgNAC* gene family is largely consistent with those identified in other species, such as Arabidopsis (117), rice (151), Chinese cabbage (188), sugarcane (88), soybean (152), durum wheat (168), and strawberry (112) [41–46]. The variation in number of genes in the *PgNAC* gene family supports the discovery of Zhang et al. [31] that the number of genes in a gene family may vary by multiple fold. The gene number variation of the *PgNAC* gene family, as demonstrated by Zhang et al. [31], may be regulated by several factors, the environmental variation of which, i.e., natural selection, may play a more important role in ginseng.

The *PgNAC* gene family is an ancient and evolutionarily inactive gene family. As the *NAC* genes identified in Arabidopsis (*AtNAC*) and rice (*OsNAC*), the *PgNAC* gene family is shown to also contain five conserved motifs (A though E), in addition to the NAC domain. Moreover, phylogenetic analysis clusters the *PgNAC* gene family into 11 subfamilies, but none of them is the *P*. *ginseng* linkage-specific or diverged after *P*. *ginseng* split from Arabidopsis. These results consistently indicate the ancient and inactive evolution nature of the *PgNAC* gene family.

Furthermore, this study, for the first time, reveals that the functions of the *PgNAC* gene family has been substantially differentiated. This finding is supported by both the lower annotation rate (48%) and functional categorization of the *PgNAC* transcripts into multiple GO categories. However, the degree of functional differentiation of the *PgNAC* gene family is much smaller than those of the *PgNBS* gene family [18], the *PgRLK* gene family [36], the *PgCYP* gene family [37], and the *AP2/ERF* gene family [47] in ginseng. Co-expression analysis, which is an indicator of functional correlation between genes, suggests that a majority of the *PgNAC* genes remain functionally correlated, in spite to their sequence divergence. Zhang et al. [48] reported that the genes controlling a polygenic trait or involved a a common biological process were several times more likely to form a co-expression network, regardless of their sequence identities and biochemical functions.

Finally, this study provides a first line of evidence that the *PgNAC* gene family also plays roles in plant response to cold stress. This result is consistent with those of the *NAC* genes obtained in other plant species, such as rice [39]. This study examined five of the 89 *PgNAC* genes that were grouped into the same group (the SNAC group) as those *NAC* genes shown to respond to abiotic stresses [39], but they may not be all genes that have such a role in the gene family. It is highly likely that more genes in the *PgNAC* gene family also play the roles in plant response to cold stress. Given that ginseng is a winter plant species to which plant response to cold stress is crucial and Oh et al. [49] revealed that the content of total saponins, the major bioactive components of ginseng, increased significantly after its plant root was treated with low temperature (4°C), additional research is deserved to identify all the gene members of the *PgNAC* gene family involved in and comprehensively understand the molecular mechanisms of the family in response to cold stress. Such research will allow not only understanding of the molecular mechanism underlying plant response to cold stress, but also provides molecular tools useful for enhanced genetic improvement in ginseng and other winter plant species.

## Supporting information

S1 Fig Phylogenetic of the *PgNAC* genes with the *NAC* genes of other plant species known to response to abiotic stresses. (PPTX)

S2 Fig The conserved motif outside the NAC domain of the *SNAC* genes in Arabidopsis, rice and ginseng. (PPTX)

S1 Table The transcript sequences of the *PgNAC* genes identified in this study. (XLSX)

S2 Table Alignment of 251 *PgNAC* transcripts to the ginseng genome, the ginseng *NAC* gene database and ginseng protein database. (XLSX)

S3 Table The cloned *NAC* genes of *Arabidopsis thaliana* (*At*) and rice (*Oryza sativ*a, *Os*) used as the evolutionary controls for phylogenetic analysis of the *PgNAC* gene family. (XLSX)

S4 Table Annotation and GO functional categorization of the *PgNAC* gene transcripts. (XLSX)

S5 Table Expressions of the *PgNAC* transcripts in 14 tissues of a four-year-old ginseng plant, four-year-old plant roots of 42 genotypes (cultivars) and the roots of four different year-old plants. (XLSX)
