# Supplementary material for: Identification, characterization and functional differentiation of the NAC gene family and its roles in response to cold stress in ginseng, Panax ginseng C.A. Meyer
Source: PLoS One. 2020 Jun 11;15(6):e0234423. doi: 10.1371/journal.pone.0234423 (PMC7289381; doi:10.1371/journal.pone.0234423)
Supplement: S1 Fig — (PPTX) [file pone.0234423.s001.pptx]

## Slide 1
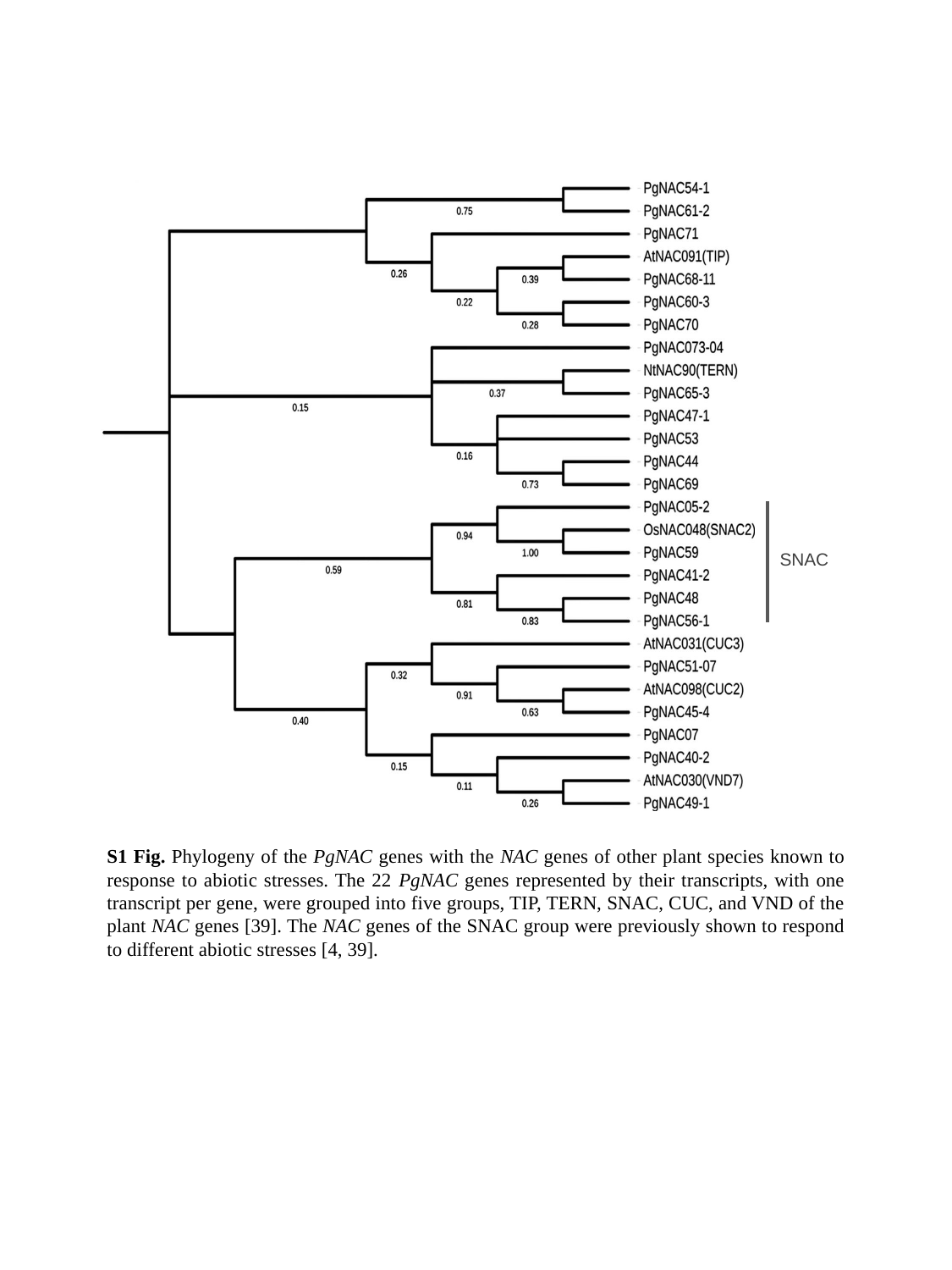

SNAC
S1 Fig. Phylogeny of the PgNAC genes with the NAC genes of other plant species known to response to abiotic stresses. The 22 PgNAC genes represented by their transcripts, with one transcript per gene, were grouped into five groups, TIP, TERN, SNAC, CUC, and VND of the plant NAC genes [39]. The NAC genes of the SNAC group were previously shown to respond to different abiotic stresses [4, 39].
