# Supplementary material for: Identification, characterization and functional differentiation of the NAC gene family and its roles in response to cold stress in ginseng, Panax ginseng C.A. Meyer
Source: PLoS One. 2020 Jun 11;15(6):e0234423. doi: 10.1371/journal.pone.0234423 (PMC7289381; doi:10.1371/journal.pone.0234423)
Supplement: S2 Fig — (PPTX) [file pone.0234423.s002.pptx]

## Slide 1
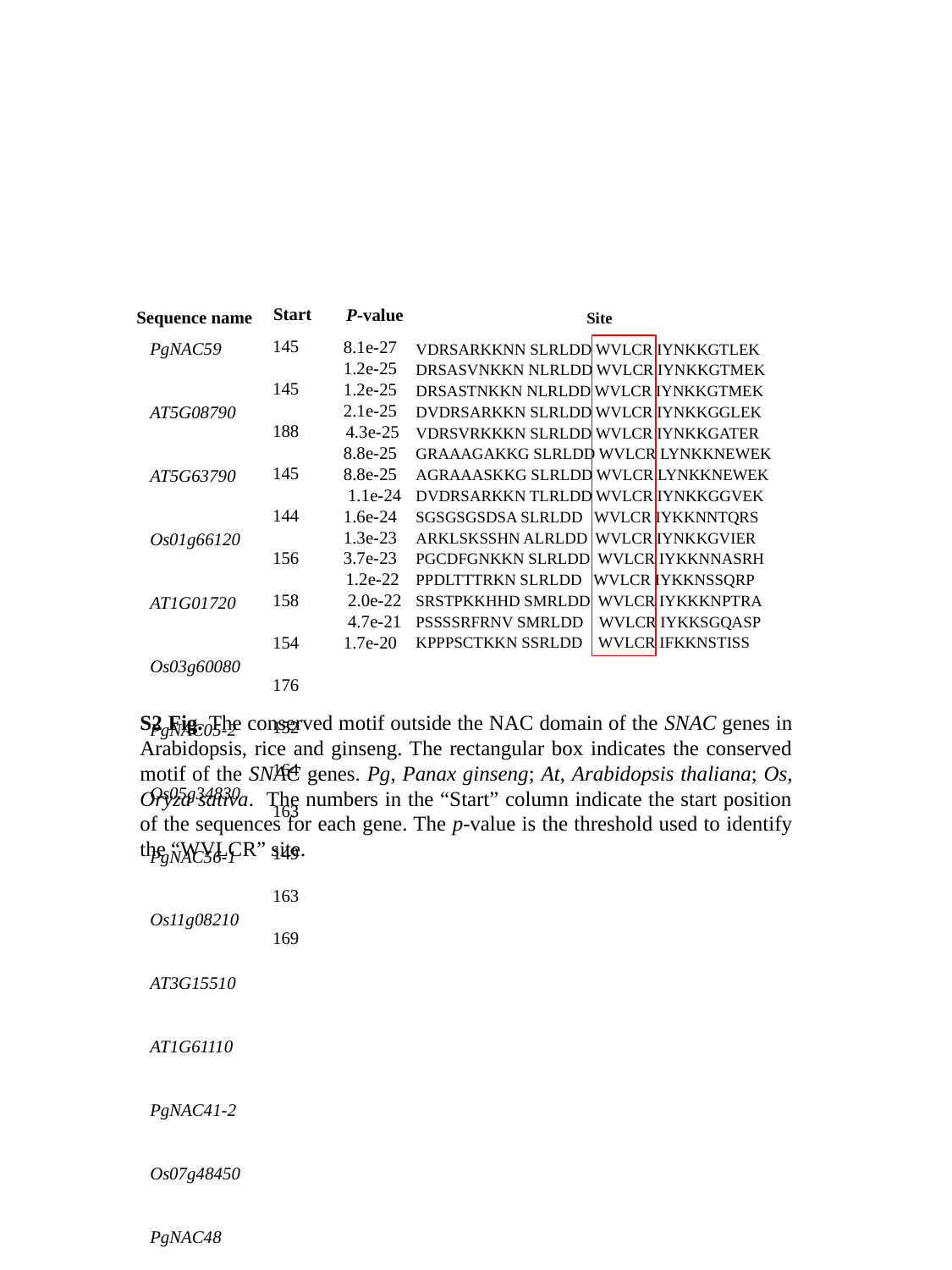

Sequence name
 PgNAC59
 AT5G08790
 AT5G63790
 Os01g66120
 AT1G01720
 Os03g60080
 PgNAC05-2
 Os05g34830
 PgNAC56-1
 Os11g08210
 AT3G15510
 AT1G61110
 PgNAC41-2
 Os07g48450
 PgNAC48
Start
145
145
188
145
144
156
158
154
176
152
164
163
149
163
169
P-value
8.1e-27
1.2e-25
1.2e-25
2.1e-25
4.3e-25
8.8e-25
8.8e-25
1.1e-24
1.6e-24
1.3e-23
3.7e-23
1.2e-22
2.0e-22 4.7e-21 1.7e-20
Site
VDRSARKKNN SLRLDD WVLCR IYNKKGTLEK DRSASVNKKN NLRLDD WVLCR IYNKKGTMEK DRSASTNKKN NLRLDD WVLCR IYNKKGTMEK DVDRSARKKN SLRLDD WVLCR IYNKKGGLEK VDRSVRKKKN SLRLDD WVLCR IYNKKGATER GRAAAGAKKG SLRLDD WVLCR LYNKKNEWEK AGRAAASKKG SLRLDD WVLCR LYNKKNEWEK DVDRSARKKN TLRLDD WVLCR IYNKKGGVEK SGSGSGSDSA SLRLDD WVLCR IYKKNNTQRS ARKLSKSSHN ALRLDD WVLCR IYNKKGVIER PGCDFGNKKN SLRLDD WVLCR IYKKNNASRH PPDLTTTRKN SLRLDD WVLCR IYKKNSSQRP SRSTPKKHHD SMRLDD WVLCR IYKKKNPTRA PSSSSRFRNV SMRLDD WVLCR IYKKSGQASP KPPPSCTKKN SSRLDD WVLCR IFKKNSTISS
S2 Fig. The conserved motif outside the NAC domain of the SNAC genes in Arabidopsis, rice and ginseng. The rectangular box indicates the conserved motif of the SNAC genes. Pg, Panax ginseng; At, Arabidopsis thaliana; Os, Oryza sativa. The numbers in the “Start” column indicate the start position of the sequences for each gene. The p-value is the threshold used to identify the “WVLCR” site.
